# Supplementary material for: Structural insights into the transporting and catalyzing mechanism of DltB in LTA D-alanylation
Source: Nat Commun. 2024 Apr 22;15:3404. doi: 10.1038/s41467-024-47783-7 (PMC11035591; doi:10.1038/s41467-024-47783-7)
Supplement: Supplementary file 1 — Supplementary Information [file 41467_2024_47783_MOESM1_ESM.pdf]

Supplementary Information

Structural Insights into the Transporting and Catalyzing Mechanism of DltB in LTA D-alanylation

Pingfeng Zhang[1, 3, *], Zheng Liu[2,3, *]

[1] Cancer Center, Renmin Hospital of Wuhan University, Wuhan, China.

[2] School of Medicine, Kobilka Institute of Innovative Drug Discovery, the Chinese University of Hong Kong (Shenzhen), Shenzhen, China

[3] These authors contribute equally.

[*]Corresponding authors:

Pingfeng Zhang: pingfeng.zhang@whu.edu.cn

Zheng Liu: liuzheng@cuhk.edu.cn

Lead contact:
Pingfeng Zhang: pingfeng.zhang@whu.edu.cn

Supplementary Fig. 1

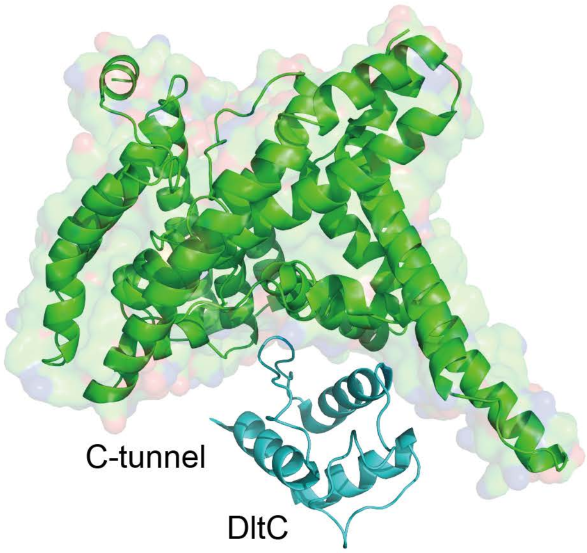

Supplementary Figure 1. C-tunnel of DltB on the cytoplasmic side.

DltC binds to monomeric DltB at the C-tunnel, generated with the crystal structure of the DltB/C complex (PDB code: 6buh). DltB is shown in green, and DltC is shown in cyan.

Supplementary Fig. 2

a

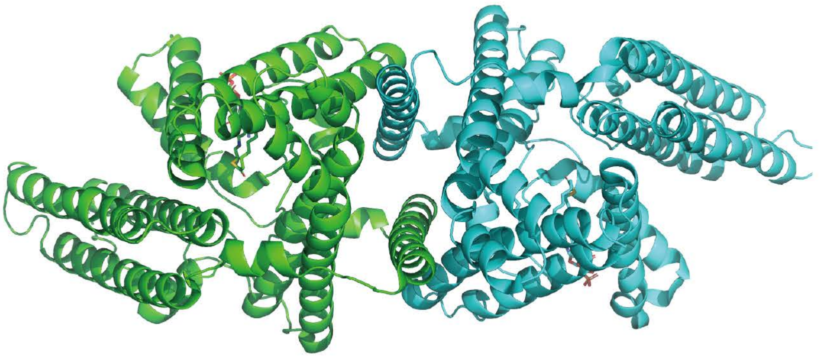

b

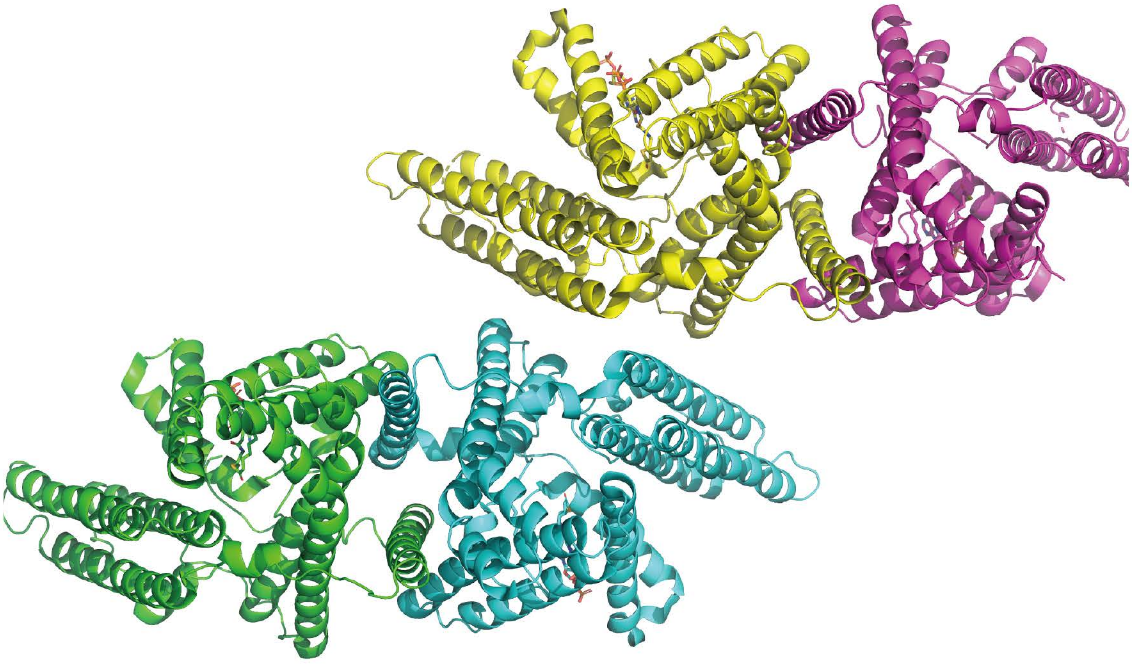

c

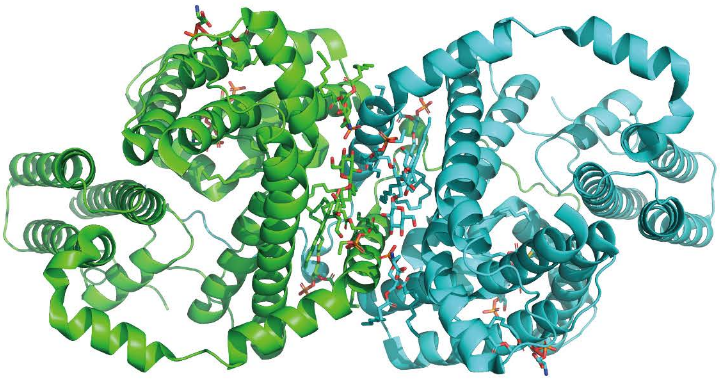

Supplementary Figure 2. Oligomeric organization of MBOAT members.

a, ACAT1 dimer generated with PDB 6p2j.

b, ACAT1 tetramer generated with PDB 6p2p.

c, DGAT1 dimer generated with PDB 6vp0.

Supplementary Fig. 3

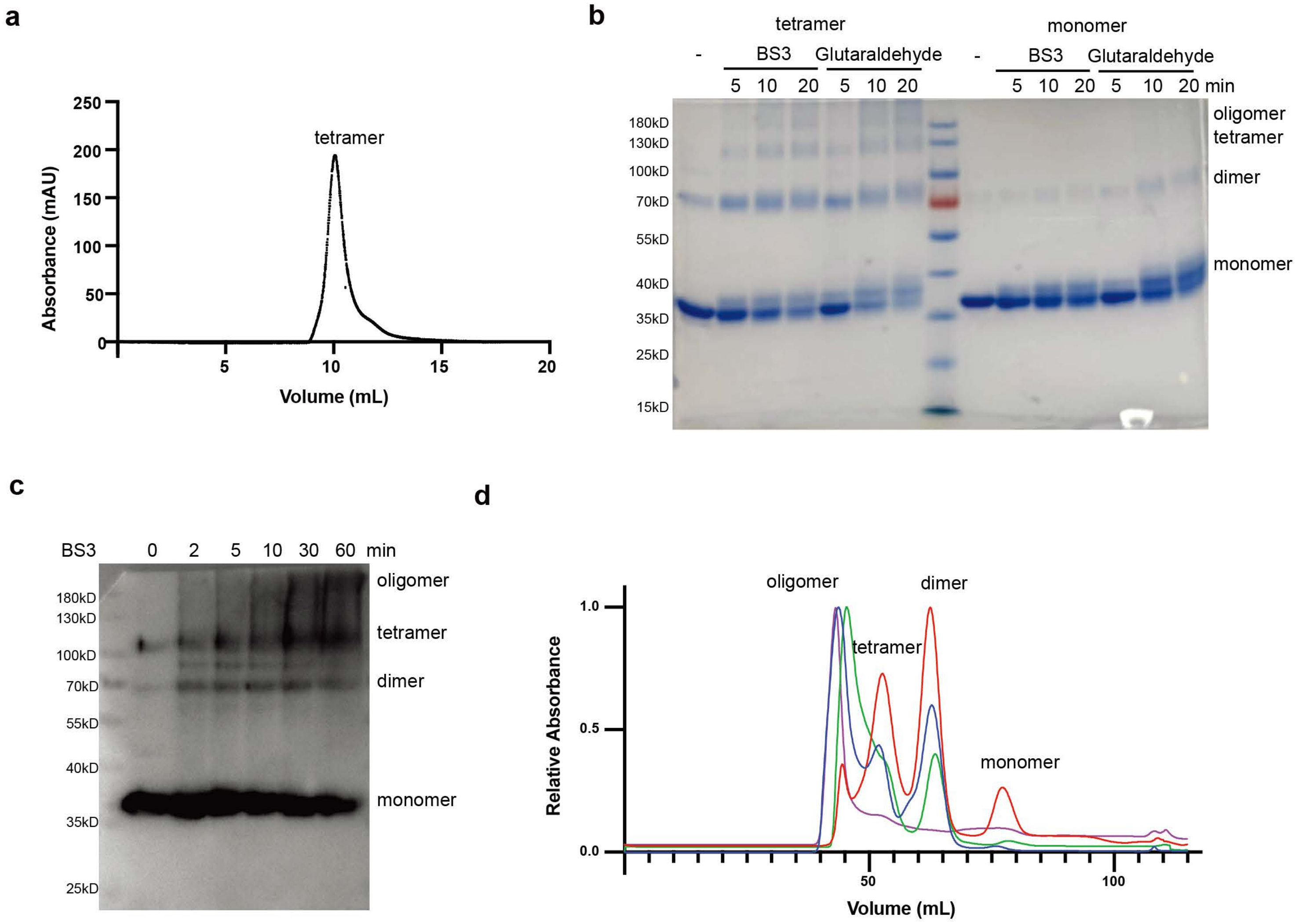

Supplementary Figure 3. Stable DltB tetramer and its dynamics during purification.

a, DltB tetramers rerun on a Superdex 200 column. The tetramer peak on the chromatogram is labeled.

b, Crosslinking of purified DltB monomer and tetramer by BS3 or glutaraldehyde. Monomers, dimers, tetramers, and oligomers are labeled on Coomassie-stained SDS-PAGE gel.

c, Crosslinking of DltB on the isolated cell membrane. DltB monomers, dimers, tetramers, and oligomers are detected via an anti-His antibody.

d, Different batches of DltB purification on a Superdex 200 column. Chromatographic peaks of DltB monomers, dimers, tetramers, and oligomers are labeled.

Supplementary Fig. 4

a, Workflow of cryo-EM image processing of the apo-DltB dataset:

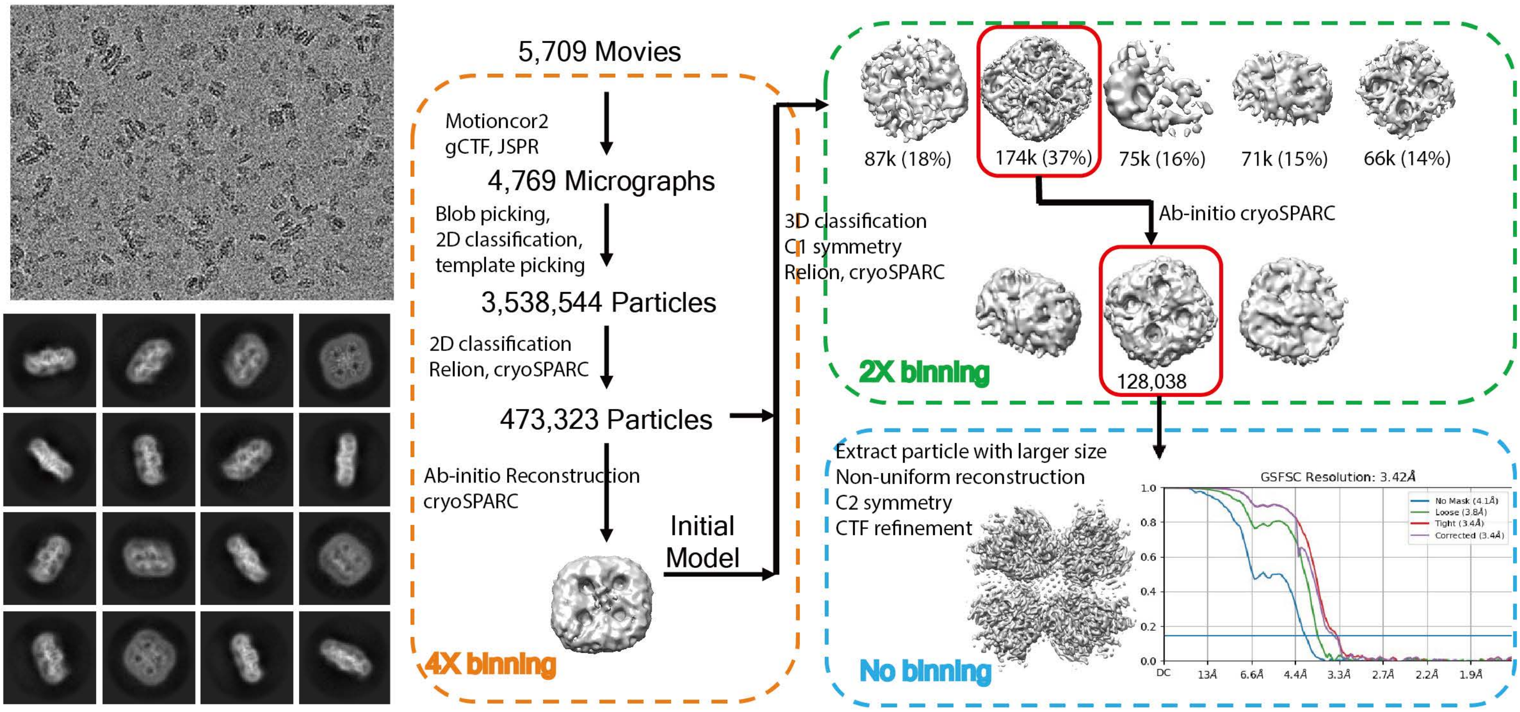

b, Local resolution distributions:

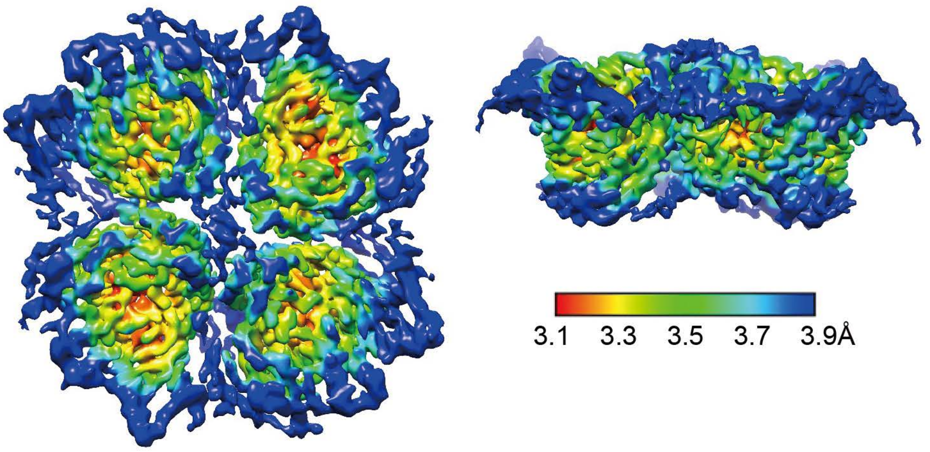

c, Angular distributions of the particles:

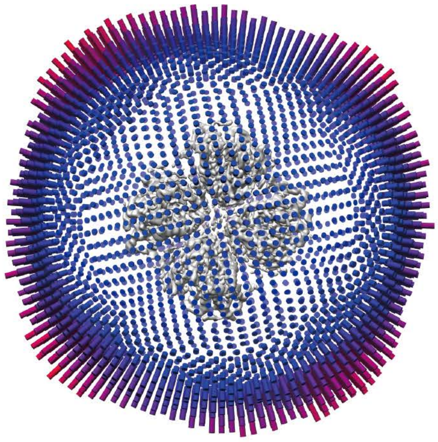

Supplementary Figure 4. Cryo-EM structure determination of apo-DltB tetramer

a, Workflow of cryo-EM image processing of the apo-DltB dataset. Top left: A representative motion-corrected cryo-electron micrograph of the apo-DltB dataset. Bottom left: Reference-free 2D class averages, highlighting clear density for tetrameric composition. The middle panel presents the preprocessing of the four-time binning images. Top right: Relion-3D classification and cryoSPARC *ab-initio* procedures to sort out different classes, with a subset of optimal or suboptimal particles selected from micrographs processed with 2-time binning images. Bottom right: A final map was reconstructed using non-binning images and colored in grey, and the FSC curves were generated including preparations with no mask, loose mask, tight mask, and the corrected way in cryoSPARC. The overall resolution, as determined by a golden standard FSC (GSFSC) cut-off value of 0.143 (purple line), is 3.42 Å.

b, Local resolution distributions for the apo-DltB structure.

c, Angular distributions of the particles used in the final reconstruction of apo-DltB.

Supplementary Fig. 5

a, Workflow of cryo-EM image processing of the DltB/DltC dataset:

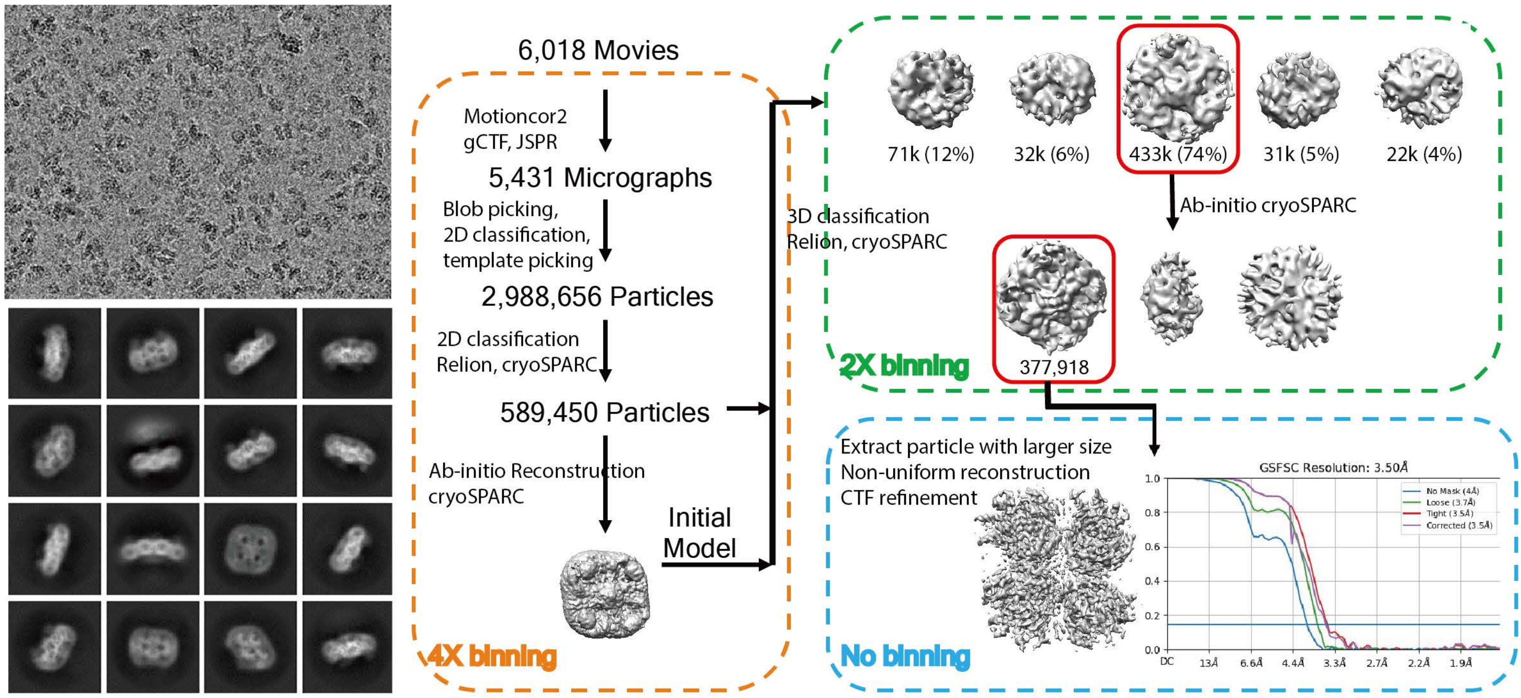

b, Local resolution distributions:

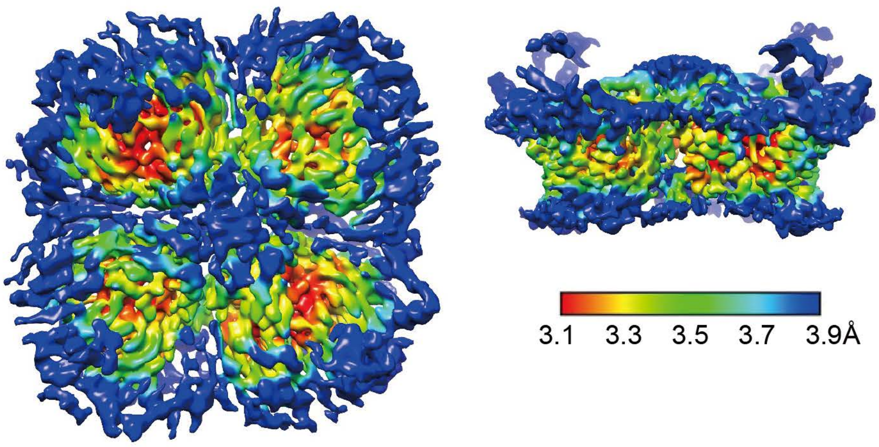

c, Angular distributions of the particles:

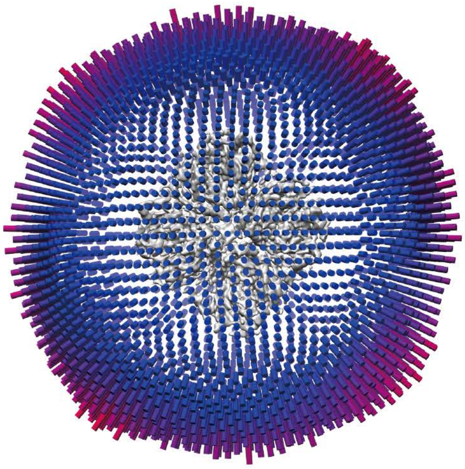

Supplementary Figure 5. Cryo-EM structure determination of DltB tetramer complexed with DltC

a, Workflow of cryo-EM image processing of the DltB in complex with DltC dataset. Top left: A representative motion-corrected cryo-electron micrograph of the DltB-DltC complex dataset. Bottom left: Reference-free 2D class averages, highlighting clear density for tetrameric composition. The middle panel presents the preprocessing of the four-time binning images. Top right: Relion-3D classification and cryoSPARC *ab-initio* procedures to sort out different classes, with a subset of optimal or suboptimal particles selected from micrographs processed with 2-time binning images. Bottom right: A final map was reconstructed using non-binned images and colored in grey, and the FSC curves were generated including preparations with no mask, loose mask, tight mask, and the corrected way in cryoSPARC. The overall resolution, as determined by a golden standard FSC (GSFSC) cut-off value of 0.143 (purple line), is 3.50 Å.

b, Local resolution distributions for the DltB/C complex structure.

c, Angular distributions of the particles used in the final reconstruction of the DltB/C complex.

Supplementary Fig. 6

a, Workflow of cryo-EM image processing of the DltB/m-AMSA dataset:

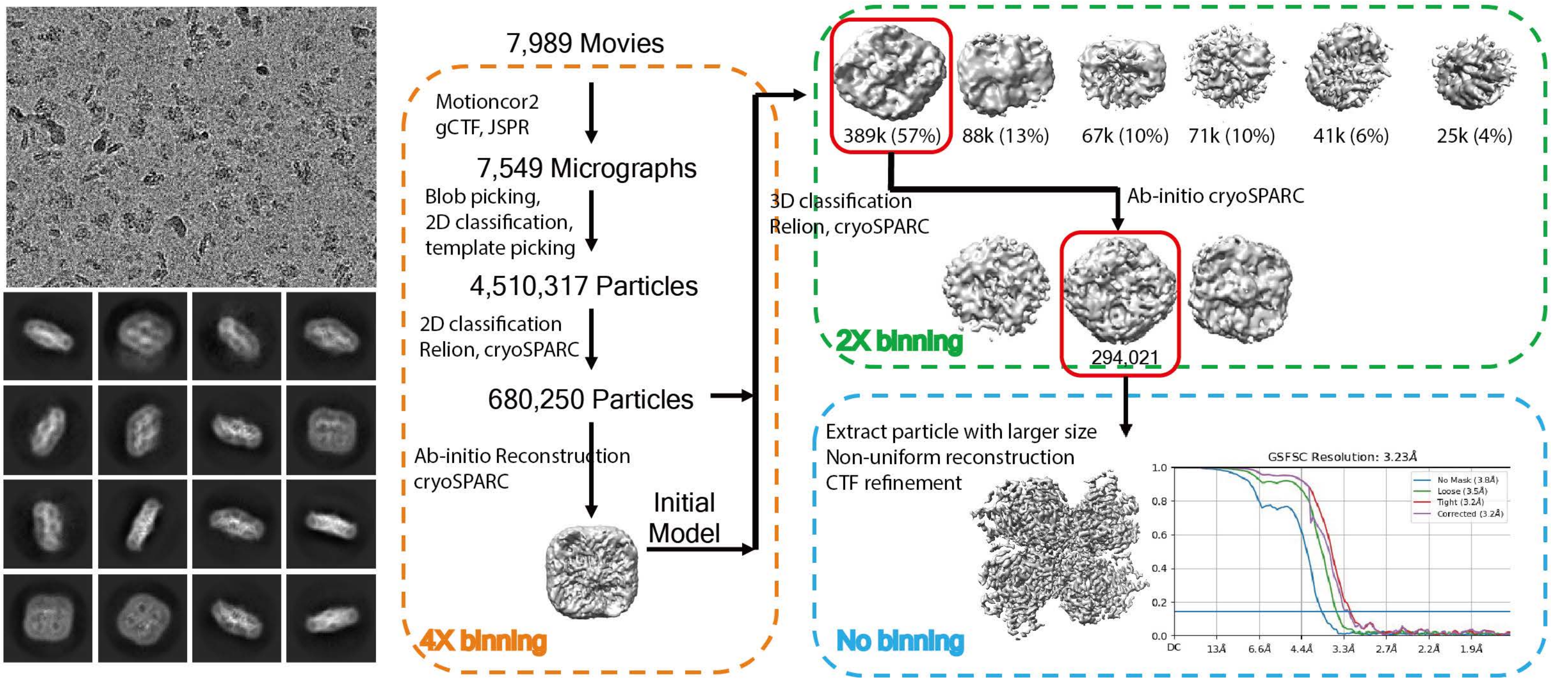

b, Local resolution distributions:

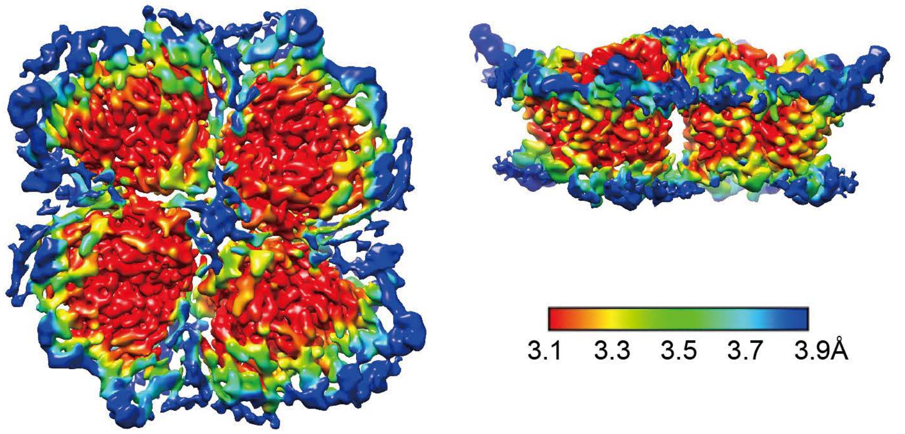

c, Angular distributions of the particles:

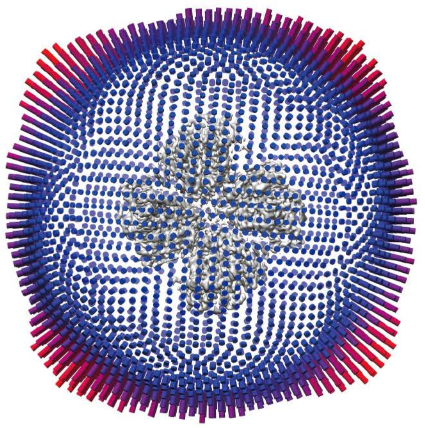

Supplementary Figure 6. Cryo-EM structure determination of DltB tetramer complexed with the inhibitor m-AMSA

a, Workflow of cryo-EM image processing of the DltB in complex with m-AMSA dataset. Top left: A representative motion-corrected cryo-electron micrograph of the DltB containing m-AMSA dataset. Bottom left: Reference-free 2D class averages, highlighting clear density for tetrameric composition. The middle panel presents the preprocessing of the four-time binning images. Top right: Relion-3D classification and cryoSPARC *ab-initio* procedures to sort out different classes, with a subset of optimal or suboptimal particles selected from micrographs processed with 2-time binning images. Bottom right: A final map was reconstructed using non-binned images and colored in grey, and the FSC curves were generated including preparations with no mask, loose mask, tight mask, and the corrected way in cryoSPARC. The overall resolution as determined by an golden standard FSC (GSFSC) cut-off value of 0.143 (purple line), is 3.23 Å.

b, Local resolution distributions for the DltB/m-AMSA structure.

c, Angular distributions of the particles used in the final reconstruction of the DltB/m-AMSA complex.

Supplementary Fig. 7

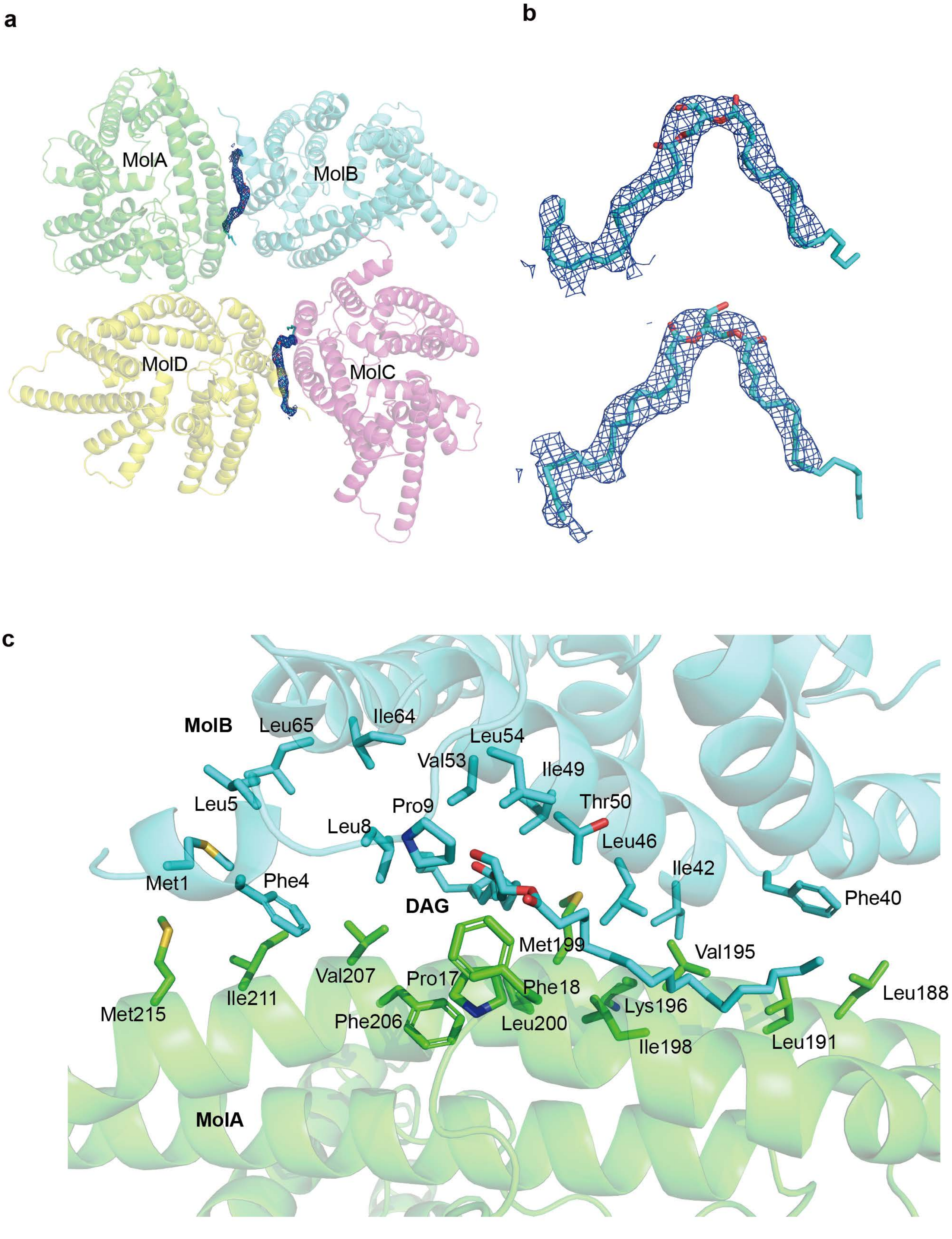

Supplementary Figure 7. Lipid molecules bound on the intratetramer interfaces of DltB

a, Overview of DAG electron density on the DltB intratetramer interface. The blue mesh represents the DAG bound on the MolA/B and MolC/D interfaces.

b, Close-up view of the density maps (contoured at 5σ level) of the two DAG molecules on the MolA/B and MolC/D interfaces.

c, DAG binding environment on MolA/B interface. The side chains of the key residues are shown as stick models and labeled.

Supplementary Fig. 8

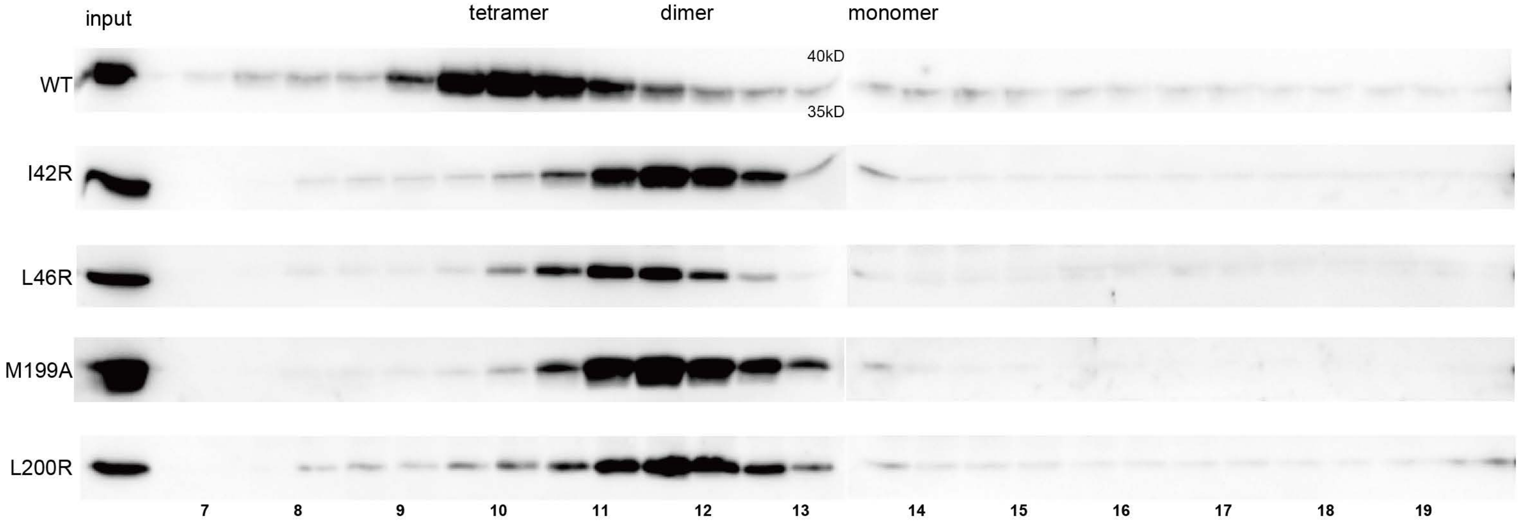

Supplementary Figure 8. Wild-type or mutant DltB proteins on gel filtration column

Membranes containing DltB WT or mutants (I42R, L46R, M199A, and L200R) were solubilized by β-DDM and run on a Superdex 200 column. Fractions containing DltB proteins were detected by western blot using an anti-His antibody. Input, tetramer, dimer, and monomer fractions are labeled. These experiments have been performed three times.

Supplementary Fig. 9

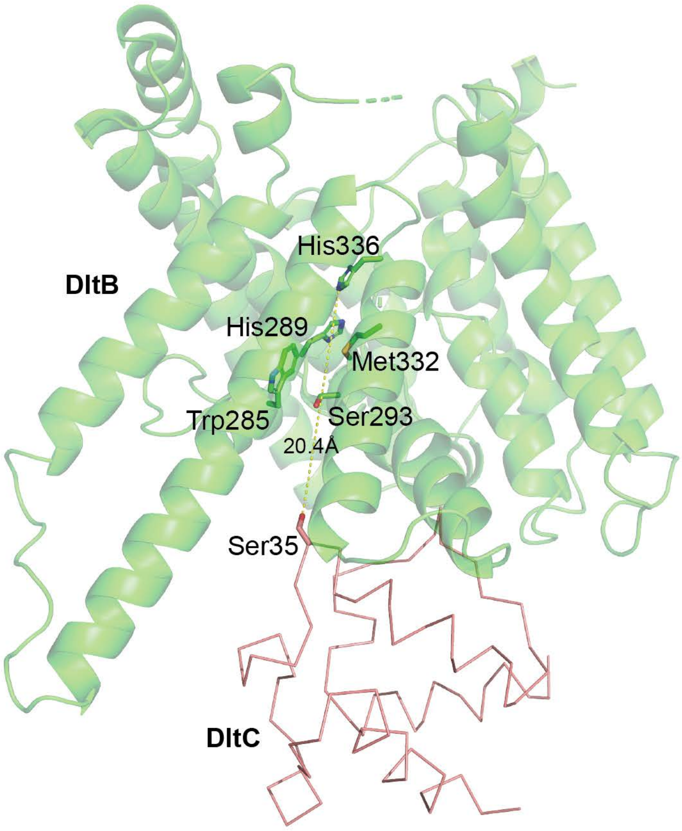

Supplementary Figure 9. DltC is able to deliver the D-alanyl group to the active site in DltB

Ser35 on DltC is 20.4 Å away from the active site residue His336 in DltB. The model is generated with the crystal structure of the DltB/C complex (PDB code: 6buh). Side chains of residues His336, His289, Met332, Trp285, and Ser293 are shown as stick models and labeled.

Supplementary Fig. 10

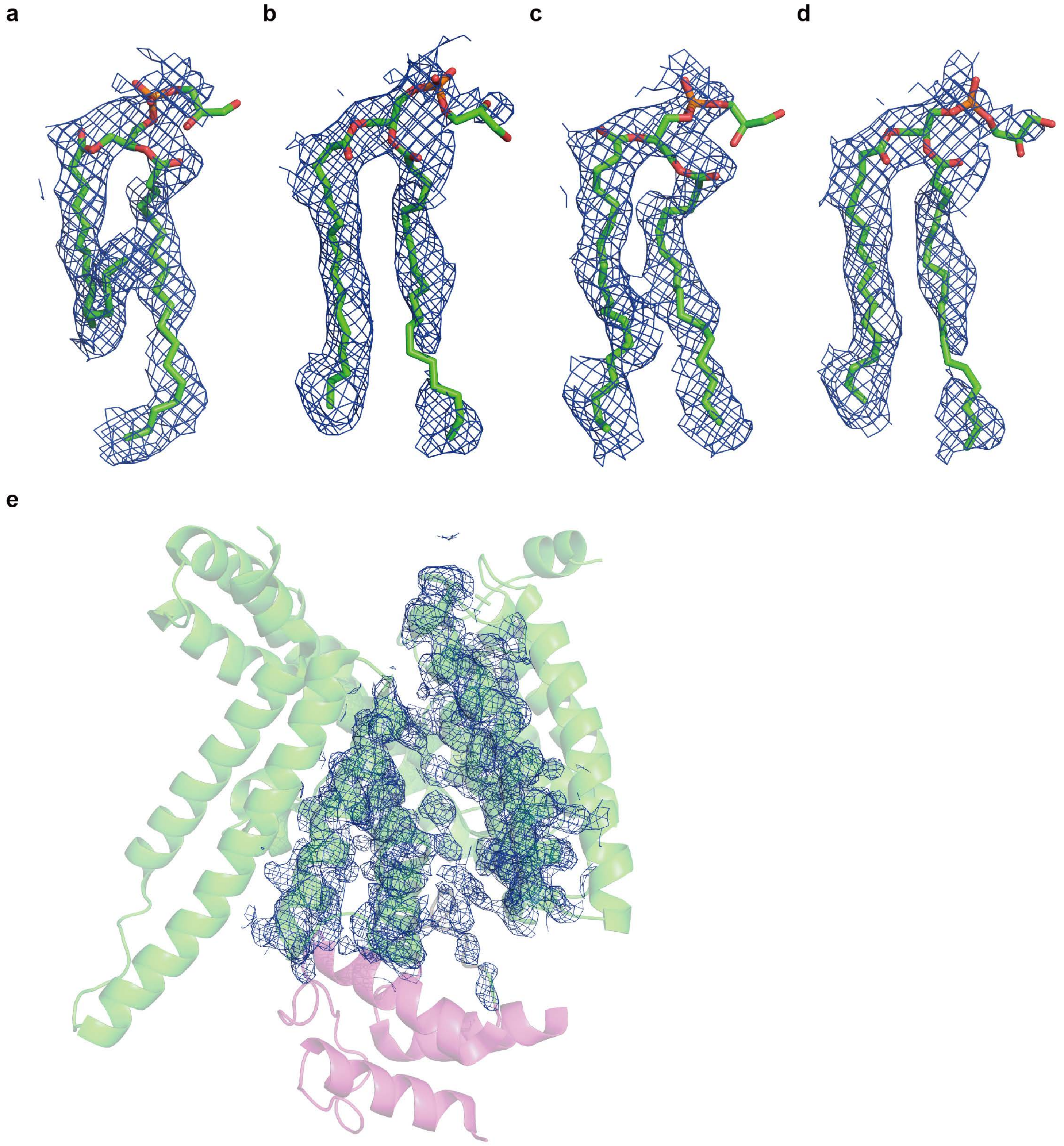

Supplementary Figure 10. Lipids bound in the substrate-binding cleft of DltB

a-d, Electron potential density maps (contoured at 5σ level) of phosphatidylglycerol bound in the substrate-binding cleft of DltB. The PG model from MolA~MolD in the DltB tetramer is generated from the cryo-EM structure of the DltB/AMSA complex. The density maps are shown in blue mesh.

e, The substrate-binding cleft in the crystal structure of DltB monomer is empty. The electron density map of the cleft region in the crystal structure (PDB code: 6buh) is presented. DltB is shown in green cartoon, and DltC is shown in red.

Supplementary Fig. 11

a

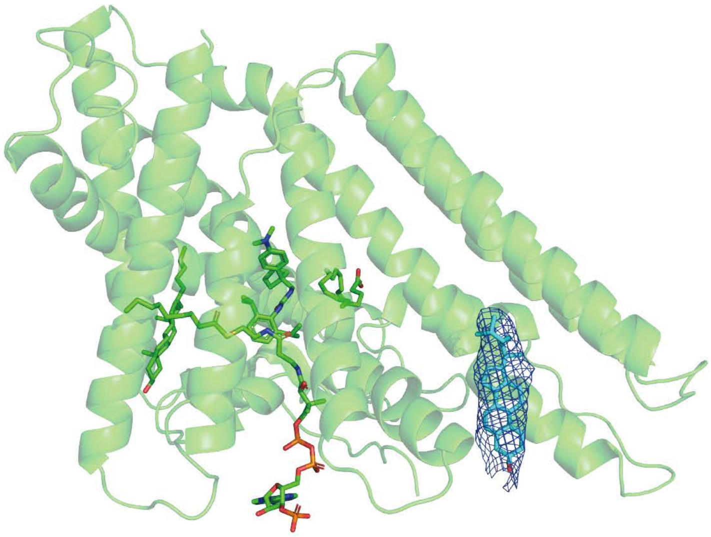

b

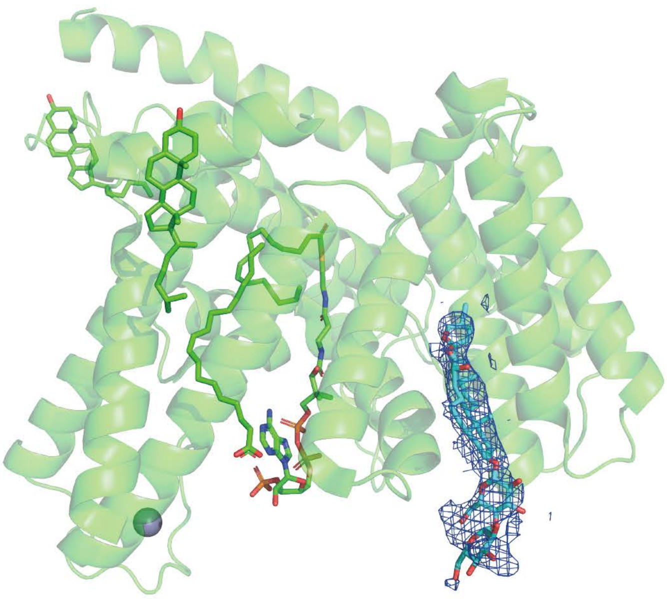

Supplementary Figure 11. The substrate binding **Cleft in other MBOAT members**

a, The electron density map of the substrate binding cleft region in the cryo-EM structure of ACAT1 (PDB code: 6vum) is presented. A cholesterol molecule is interpreted in the cleft, and the corresponding density map is shown in blue mesh.

b, A detergent digitonin molecule was assigned to the according cleft region of cryo-EM structure of PORCN (PDB code: 7ura). The density map for digitonin is presented as blue mesh.

Supplementary Table 1 | Summary of cryo-EM data collection, processing and structure refinement

	DltB apo	DltB+DltC	DltB+m-AMSA
Data collection and processing	8JES	8JF2	8JEM
Magnification	105 000	105 000	105 000
Voltage (kV)	300	300	300
Electron exposure (e-/ Å^2)	55	55	55
Detector	K3 Direct Detection Cameras		
Defocus range(μm)	[-1.7,-1.1]	[-1.7,-1.2]	[-1.8,-1.2]
Pixel size (Å)	0.83	0.83	0.83
Number of Movies (no.)	5,709	6,018	7,989
Initial particle numbers (no.)	3,538,544	2,988,656	4,510,317
Final reconstruction particle numbers (no.)	128,038	377,918	294,021
Map resolution (Å)	3.42	3.50	3.23
FSC threshold	0.143	0.143	0.143
Symmetry imposed	C2	C2	C2
Map resolution range (Å)	3.4～3.8	3.4~3.9	3.1~3.5
Refinement			
Model resolution (Å)	3.42	3.5	3.23
FSC threshold	0.5	0.5	0.5
Model resolution range (Å)	3.4～3.8	3.4~3.9	3.1~3.5
Map sharpening *B* factor (Å^2)	110.9	127.5	112.4
Model composition			
Non-hydrogen atoms	15541	17676	15930
Protein residues	1674	1990	1674
Ligands	6 PGT, 2 DGA, 35 LMT	6 PGT, 2 DGA, 25 LMT	4 ASW, 8 PGT, 2 DGA, 40 LMT
B factors (Å^2)			
Protein	148.8	102.6	90.2
Ligand	189.0	132.0	114.2
R.m.s. deviations			
Bond lengths (Å)	0.003	0.005	0.005
Bond angles (°)	0.551	0.573	0.602
Validation			
MolProbity score	2.14	2.23	2.07
Clashscore	7.32	8.91	9.05
Poor rotamer (%)	4.02	3.66	2.08
Ramachandran plot			
Favored (%)	95.92	95.34	94.96
Allowed (%)	4.02	4.66	4.98
Disallowed (%)	0.06	0.00	0.06

Supplementary Table 2 | The names of Lipids identified from DltB sample and their abbreviations.

Abbreviations	Lipids' Identity
CE	Cholesterol
CL	Cardiolipins
DAG	diacylglycerols
DGDG	digalactosyl diacylglycerols
MG	monogalactosyl
MGDG	monogalactosyl diacylglycerols
PA	phosphatidic acids
PC	phosphatidylcholines
PE	phosphatidylethanolamines
PG	phosphatidylglycerols
PI	phosphatidylinositols
PS	phosphatidylserines
SL	sphingolipids
SM	sphingomyelin
ST	Sterol lipids
TG	triacylglycerols
FA	fatty acids

Source Data for Supplementary Fig. 3b

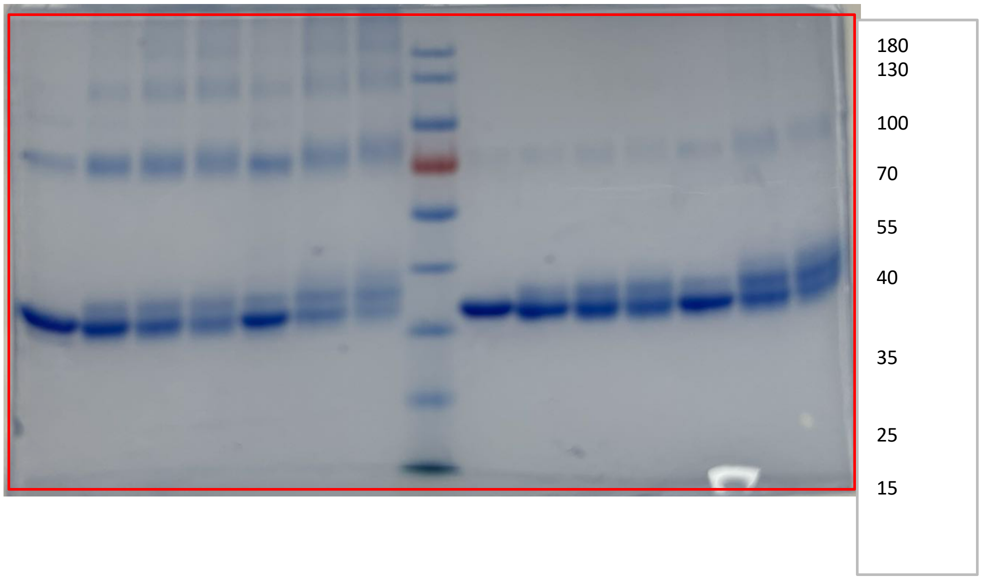

Source Data for Supplementary Fig. 3c

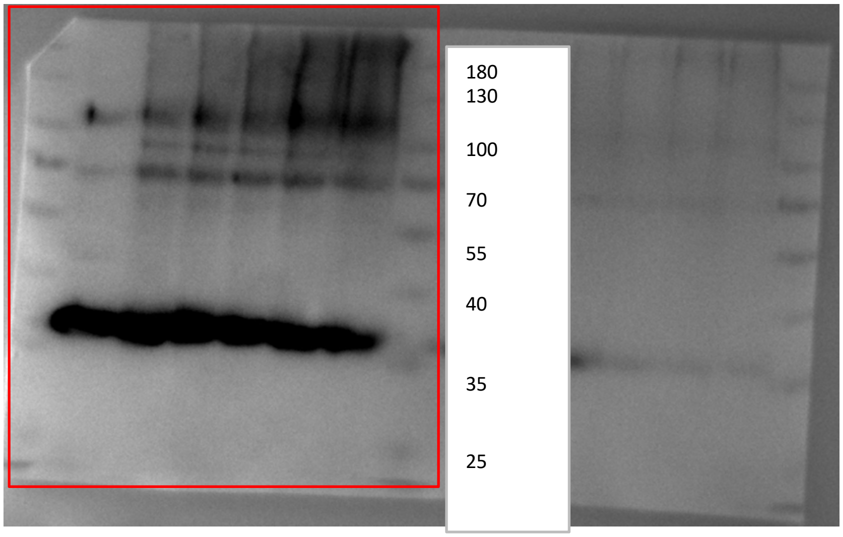

Source Data for Supplementary Fig. 8

WT

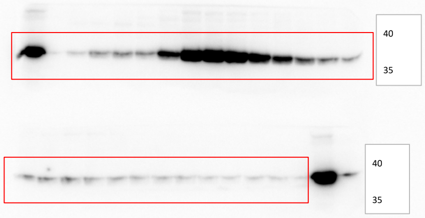

I42R

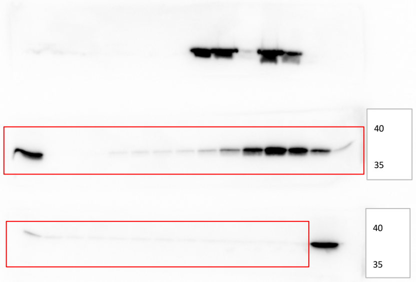

L46R

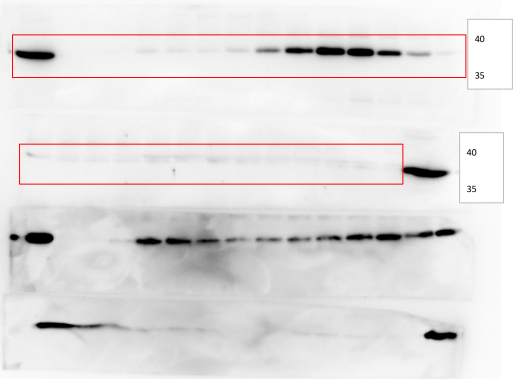

M199A

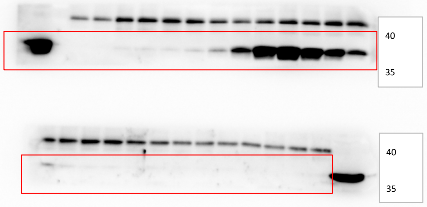

the column was contaminated.

L200R

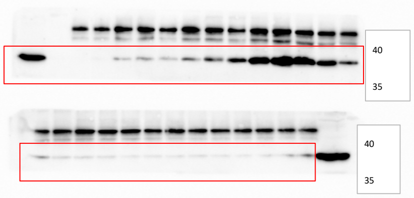

the column was contaminated.
