# Supplementary material for: Structural insights into the transporting and catalyzing mechanism of DltB in LTA D-alanylation
Source: Nat Commun. 2024 Apr 22;15:3404. doi: 10.1038/s41467-024-47783-7 (PMC11035591; doi:10.1038/s41467-024-47783-7)
Supplement: Supplementary file 3 — Description of Additional Supplementary Files [file 41467_2024_47783_MOESM3_ESM.pdf]

Description of Additional Supplementary Files

File Name: Supplementary Data 1
Description: Lipids identification and classification
The lipid species identified by mass spectrometry from four samples (cell membrane, crude DltB, DltB tetramer, DltB monomer) have been listed, along with the classification of the identified lipids in each sample.
