# Supplementary material for: Structural insights into the transporting and catalyzing mechanism of DltB in LTA D-alanylation
Source: Nat Commun. 2024 Apr 22;15:3404. doi: 10.1038/s41467-024-47783-7 (PMC11035591; doi:10.1038/s41467-024-47783-7)
Supplement: Supplementary file 5 — Reporting Summary [file 41467_2024_47783_MOESM5_ESM.pdf]

nature portfolio

Corresponding author(s): Pingfeng Zhang

Last updated by author(s): Sep 28, 2023

Reporting Summary

Nature Portfolio wishes to improve the reproducibility of the work that we publish. This form provides structure for consistency and transparency in reporting. For further information on Nature Portfolio policies, see our Editorial Policies and the Editorial Policy Checklist.

Statistics

For all statistical analyses, confirm that the following items are present in the figure legend, table legend, main text, or Methods section.

n/a	Confirmed	
[ ]	[x]	The exact sample size (*n*) for each experimental group/condition, given as a discrete number and unit of measurement
[ ]	[x]	A statement on whether measurements were taken from distinct samples or whether the same sample was measured repeatedly
[x]	[ ]	The statistical test(s) used AND whether they are one- or two-sided<br>*Only common tests should be described solely by name; describe more complex techniques in the Methods section.*
[x]	[ ]	A description of all covariates tested
[x]	[ ]	A description of any assumptions or corrections, such as tests of normality and adjustment for multiple comparisons
[ ]	[x]	A full description of the statistical parameters including central tendency (e.g. means) or other basic estimates (e.g. regression coefficient) AND variation (e.g. standard deviation) or associated estimates of uncertainty (e.g. confidence intervals)
[x]	[ ]	For null hypothesis testing, the test statistic (e.g. *F*, *t*, *r*) with confidence intervals, effect sizes, degrees of freedom and *P* value noted<br>*Give P values as exact values whenever suitable.*
[x]	[ ]	For Bayesian analysis, information on the choice of priors and Markov chain Monte Carlo settings
[x]	[ ]	For hierarchical and complex designs, identification of the appropriate level for tests and full reporting of outcomes
[x]	[ ]	Estimates of effect sizes (e.g. Cohen's *d*, Pearson's *r*), indicating how they were calculated

Our web collection on statistics for biologists contains articles on many of the points above.

Software and code

Policy information about availability of computer code

Data collection	Unicorn (Cytiva Life Science, V7.0), SerialEM (v3.80), Analyst TF (v1.7.1),
Data analysis	Relion (v3.1), MotionCor2 (v2), CTFFind4 (v4), cryoSPARC (v3), jspr (2204), gCTF (v1.06), ChimeraX (v1.6.1), Coot (v0.9.4.1), Phenix (v1.20), AnalysisBaseFileConverter, MS-DIAL (v4.70), Adobe illustrator (v24.1.3), Pymol (v2.6.0)

For manuscripts utilizing custom algorithms or software that are central to the research but not yet described in published literature, software must be made available to editors and reviewers. We strongly encourage code deposition in a community repository (e.g. GitHub). See the Nature Portfolio guidelines for submitting code & software for further information.

Data

Policy information about availability of data

All manuscripts must include a data availability statement. This statement should provide the following information, where applicable:

- Accession codes, unique identifiers, or web links for publicly available datasets
- A description of any restrictions on data availability
- For clinical datasets or third party data, please ensure that the statement adheres to our policy

The atomic coordinates generated in this study have been deposited in the Protein Data Bank under the accession numbers 8JES [https://doi.org/10.2210/pdb8jes/pdb] (apo), 8JF2 [https://doi.org/10.2210/pdb8jf2/pdb] (DltB/C complex), and 8JEM [https://doi.org/10.2210/pdb8jem/pdb] (DltB/m-AMSA complex). The cryo-EM electron potential density maps generated in this study have been deposited in the Electron Microscopy Data Bank (EMDB) under the accession numbers EMD-36194 [https://www.ebi.ac.uk/emdb/search/?q=EMD-36194] (apo), EMD-36207 (DltB/C comples) [https://www.ebi.ac.uk/emdb/search/?q=EMD-36207], and

EMD-36192 [https://www.ebi.ac.uk/emdb/search/?q=EMD-36192] (DltB/m-AMSA complex). The model of the DltB monomer (6BUH [https://www.rcsb.org/structure/6BUH]) was obtained from the PDB data bank. Uncropped scans of blots and gels in Figs. 1b-d, and 4c are supplied as Source Data. These presented in Supplementary figures are provided at the end of the Supplementary Information. Lipidomics raw data generated in this study have been uploaded in MetaboLights under accession number MTBLS9864 (https://www.ebi.ac.uk/metabolights/editor/study/MTBLS9864). All other data are available from the corresponding authors upon request.

Research involving human participants, their data, or biological material

Policy information about studies with human participants or human data. See also policy information about sex, gender (identity/presentation), and sexual orientation and race, ethnicity and racism.

Reporting on sex and gender	There were no human research participants in this study.
Reporting on race, ethnicity, or other socially relevant groupings	There were no human research participants in this study.
Population characteristics	There were no human research participants in this study.
Recruitment	There were no human research participants in this study.
Ethics oversight	There were no human research participants in this study.

Note that full information on the approval of the study protocol must also be provided in the manuscript.

Field-specific reporting

Please select the one below that is the best fit for your research. If you are not sure, read the appropriate sections before making your selection.

☒ Life sciences ☐ Behavioural & social sciences ☐ Ecological, evolutionary & environmental sciences

For a reference copy of the document with all sections, see nature.com/documents/nr-reporting-summary-flat.pdf

Life sciences study design

All studies must disclose on these points even when the disclosure is negative.

Sample size	No any statistical methods were used to predetermine the sample size.<br>For negative stain electron microscopy, two to three micrographs from different locations on a single grid were recorded and examined per condition, as is common in the field. For cryo-EM structure determination, the number of movies collected and particles included in the processing workflow are listed in Extended Data Table 1. In brief, 5709 movies were collected for apo-DltB, 6018 movies for DltB/C complex, and 7989 movies for DltB/AMSA complex. Additionally, for each dataset, 128038, 377918, and 294021 final particle numbers were used for reconstitution, respectively.
Data exclusions	Particles that did not contribute to high resolution structure determination were excluded during 2D and 3D classification following the standard procedures in cryoEM data analysis, because it is essential to optimize the best particles for high-resolution map generation.
Replication	The purification of DltB protein was performed more than 30 times, all attempts without accident were successful. Cross-link assay and lysozyme-sensitivity assay have been repeated three times. All attempts at replication were successful.
Randomization	For cryo-EM data processing, the datasets were randomly split into two halves for structure determination. Randomization is unnecessary and not applicable for biochemical assays in this study.
Blinding	Blinding is unnecessary and not applicable for the biochemical assays and cryo-EM structure determination in this study.

Reporting for specific materials, systems and methods

We require information from authors about some types of materials, experimental systems and methods used in many studies. Here, indicate whether each material, system or method listed is relevant to your study. If you are not sure if a list item applies to your research, read the appropriate section before selecting a response.

Materials & experimental systems

n/a	Involved in the study
☐	☒ Antibodies
☒	☐ Eukaryotic cell lines
☒	☐ Palaeontology and archaeology
☒	☐ Animals and other organisms
☒	☐ Clinical data
☒	☐ Dual use research of concern
☒	☐ Plants

Methods

n/a	Involved in the study
☒	☐ ChIP-seq
☒	☐ Flow cytometry
☒	☐ MRI-based neuroimaging

Antibodies

Antibodies used	Anti-Hexa Histidine tag Mouse McAb (1:2000 dilution) (ProteinTech, #66005-1-Ig) and HRP-linked anti-mouse IgG antibody (1:5000 dilution) (Cell Signalling Technology Catalog #7076) were used for detection of recombinant His-DltB by Western blot.
Validation	These antibodies were validated by the manufacturers and have been used in numerous previous studies that can be found on the manufacturer's websites (https://www.ptglab.com/products/His-Tag-Antibody-66005-1-Ig.htm, https://www.cellsignal.com/products/secondary-antibodies/anti-mouse-igg-hrp-linked-antibody/7076?_requestid=4699412).
